# Effects of Biogenic Zinc Oxide Nanoparticles on Growth and Oxidative Stress Response in Flax Seedlings vs. In Vitro Cultures: A Comparative Analysis

**DOI:** 10.3390/biom10060918

**Published:** 2020-06-17

**Authors:** Afifa Zaeem, Samantha Drouet, Sumaira Anjum, Razia Khurshid, Muhammad Younas, Jean Philippe Blondeau, Duangjai Tungmunnithum, Nathalie Giglioli-Guivarc’h, Christophe Hano, Bilal Haider Abbasi

**Affiliations:** 1 Department of Biotechnology, Quaid-i-Azam University, Islamabad 45320, Pakistan; afifa.zaeem@vu.edu.pk (A.Z.); raziakhurshid666@gmail.com (R.K.); pk.younas@gmail.com (M.Y.); 2 Department of Biotechnology, Virtual University of Pakistan, Rawalpindi Campus 46300, Pakistan; 3 Laboratoire de Biologie des Ligneux et des Grandes Cultures (LBLGC), INRAE USC1328, University of Orleans, F28000 Chartres, France; samantha.drouet@univ-orleans.fr (S.D.); duangjai.tun@mahidol.ac.th (D.T.); 4 Department of Biotechnology, Kinnaird College for Women, Lahore 54000, Pakistan; sumaira.anjum@kinnaird.edu.pk; 5 Conditions Extrêmes et Matériaux, Haute Température et Irradiation (CEMHTI) CNRS UPR3079, 1D Avenue de la Recherche Scientifique, 45071 Orléans, France; jean-philippe.blondeau@univ-orleans.fr; 6 Faculty of Pharmacy, Department of Pharmaceutical Botany, Mahidol University, Bangkok 10400, Thailand; 7 Biomolecules et Biotechnologies Vegetales, EA2106, Universite Francois-Rabelais de Tours, 37000 Tours, France; nathalie.guivarch@univ-tours.fr

**Keywords:** *Linum usitatissimum* L., zinc oxide nanoparticles, antioxidants, peroxidase, superoxide dismutase, phenolics, flavonoids, lignans, neolignans

## Abstract

*Linum usitatissimum* biosynthesizes lignans and neolignans that are diet and medicinally valuable metabolites. In recent years, zinc oxide nanoparticles (ZnONPs) have emerged as potential elicitors for the enhanced biosynthesis of commercial secondary metabolites. Herein, we investigated the influence of biogenic ZnONPs on both seedlings and stem-derived callus of *L. usitatissimum*. Seedlings of *L. usitatissimum* grown on Murashige and Skoog (MS) medium supplemented with ZnONPs (1–1000 mg/L) presented the highest antioxidant activity, total phenolic content, total flavonoid content, peroxidase and superoxide dismutase activities at 500 mg/L, while the maximum plantlet length was achieved with 10 mg/L. Likewise, the high-performance liquid chromatography (HPLC) analysis revealed the enhanced production of secoisolariciresinol diglucoside, lariciresinol diglucoside, dehydrodiconiferyl alcohol glucoside and guaiacylglycerol-β-coniferyl alcohol ether glucoside in the plantlets grown on the 500 mg/L ZnONPs. On the other hand, the stem explants were cultured on MS media comprising 1-naphthaleneacetic acid (1 mg/L) and ZnONPs (1–50 mg/L). The highest antioxidant and other activities with an enhanced rooting effect were noted in 25 mg/L ZnONP-treated callus. Similarly, the maximum metabolites were also accumulated in 25 mg/L ZnONP-treated callus. In both systems, the dose-dependent production of reactive oxygen species (ROS) was recorded, resulting in oxidative damage with a more pronounced toxic effect on in vitro cultures. Altogether, the results from this study constitute a first comprehensive view of the impact of ZnONPs on the oxidative stress and antioxidant responses in seedlings vs. in vitro cultures.

## 1. Introduction

Flax (*Linum usitatissimum* L.) belongs to the *Linaceae* family. This plant is one of the oldest crops being cultivated on a large area [1]. Flax has a wide range of usage in the food, paint and textile industries. It is also used as medicinal remedy for cough, fatigue, ageing, inflammation, diabetes, cancers and cardiovascular abnormalities [2,3]. Its polyphenols, fatty acids, vitamins and cyclic peptides are some examples of the medicinally significant compounds present in *L. usitatissimum*. Among them, one of the most significant are lignans and neolignans [4]. Beside their pronounced antioxidant action [5,6], upon ingestion, plant lignans are metabolized in the intestines by microbiota into enterolignans, which display anti-cancer actions against breast, intestine and prostate cancers [7,8]. Because of these health-promoting metabolites, *L. usitatissimum* represents an attractive crop and medicinal plant [9,10,11,12].

Nanoparticles (NPs) are one of the emerging threats to the plant environment [13]. Plants are perceived as abiotic elicitors that stimulate the increased production of secondary metabolites [13]. For instance, in our previous reports, chemogenic silver nanoparticles have been able to stimulate the production of lignans and neolignans in flax [14,15]. It is accepted that NPs may have both beneficial and harmful effects on plants, in particular depending on the concentration [16]. Many reports have shown that when used at a lower concentration, NPs can promote plant growth and the production of secondary metabolites [17]. It may be due to NP interaction with some of the plant cell wall and membrane components [17]. The structure of the plant cell wall is consistent with the size of the NPs for entry into the cell where the reactive oxygen species (ROS) accumulation can be triggered [18]. In turn, ROS can interfere with the plasma membrane and affect the permeability of the cells. As a result, even more NPs can reach the cells that cause severe stress, which can stimulate the production of stress-induced secondary metabolites [19]. Metals are known to influence plant growth, physiological and biochemical profiles and seed germination process [20]. Zn is considered to be the essential metal for plant growth, but may be a phytotoxic metal at a critical concentration depending on the considered plant tissue and/or species [21]. If the concentration of Zn exceeds this tolerance limit, it accumulates in tissues and causes phytotoxic effects, resulting in higher ROS production [22] and limited growth [23]. Accordingly, the concentration of Zn provided to plants should be precisely controlled for optimal plant development [24]. On the contrary, zinc oxide nanoparticles (ZnONPs) elicitation capability has been used to promote the production of bioactive secondary metabolites in medicinal plants [25,26].

Oxidative stress induction is a common response of plants to nanoparticle (NP) exposure because of their toxicity, but also as an inductive signal for the production of antioxidant secondary metabolites [17]. Enzymatic antioxidant response also contributes to the control of oxidative stress induced by NPs. The partition between enzymatic vs. non-enzymatic antioxidant responses in plants subjected to NP treatment is still unclear, and their comparison to the whole plant vs. in vitro culture levels is desired. Herein, we investigated the influence of biogenically obtained ZnONPs [27] on flax seedlings vs. callus by monitoring the growth, oxidative stress markers and enzymatic vs. non-enzymatic antioxidant responses. A first comprehensive view of the impact of ZnONPs on the oxidative stress and antioxidant responses in seedlings vs. in vitro cultures is presented.

## 2. Materials and Methods

### 2.1. Chemicals

All the reagents used in experimentation were from Sigma-Aldrich and Merck (Saint Quentin Fallavier, France).

### 2.2. Plant Materials

Seeds of flax (*Linum usitatissimum* L., commercial cultivar Barbara) were bought from Swat Collection (Swat, Pakistan). A voucher specimen of the plant was deposited in the herbarium of Department of Biotechnology, Quaid-i-Azam University (Islamabad, Pakistan) under voucher ID: BHA-LuBS2018#1.

### 2.3. Preparation and Characterization of Bio-Assisted ZnONPs

The bio-assisted synthesis of ZnONPs and their complete characterization are described in our previous work [27]. Briefly, the plant extract (1 mL) and a 0.02 M solution Zn (O_2_CCH_3_)_2_ (H_2_O) (50 mL) were constantly stirred under the continuous dropwise addition of 2 M NaOH until the solution retained pH 12. White precipitates were formed, which were rinsed off in distilled water followed by drying in oven at 60 °C for 24 h. The X-ray diffraction analysis (XRD) was done using the XRD instrument (AXS DS Advance, Bruker, Billerica, MA, USA), which has a cathode ray emitting X-rays on samples, and the size of ZnONPs was calculated with the Debye–Scherrer formula. Furthermore, to determine the major functional groups in ZnONPs, Fourier-transform infrared spectroscopy (FTIR, Bruker, Billerica, MA, USA) was employed in the spectral array from 400 to 4000 cm^−1^ [27].

### 2.4. Medium Preparation and Inoculation for Seeds

The germination medium was prepared in 100 mL Erlenmeyer flask derived from Murashige and Skoog medium (pH 5.6) supplemented with 30 g/L sucrose and 8 g/L agar [28,29]. This medium was further supplemented with ZnONPs at concentrations of 1, 10, 100, 500 and 1000 mg/L and sonicated for 5 min (Elmasonic E 30 H; 37 kHz, Elma Schmidbauer GmbH, Singen, Germany) to spread nanoparticles equally. The *L. usitatissimum* seeds were sterilized as described previously [30] before transferring to the prepared medium containing nanoparticles. Each flask was inoculated with five viable seeds and transferred to a climatic growth chamber at 25 ± 1 °C temperature, 45–50 μmol/m^2^/s total amount of photosynthetically active radiation, 16/8 h light/dark cycle, and 30% relative humidity for 13 days. Afterward, the seedlings were harvested and analyzed for growth and biochemical markers.

### 2.5. Medium Preparation and Inoculation of Stem-Derived Callus

Callus medium was prepared in 100 mL Erlenmeyer flask derived from the Murashige and Skoog medium, pH 5.6, supplemented with 30 g/L sucrose, 1 mg/L 1-naphthaleneacetic acid NAA and 8 g/L agar [28,31]. This medium was further supplemented with ZnONPs at concentrations of 5, 10, 25, 35, 50 mg/L as described above. For the inoculation of stem explants, about 6 mm long pieces of stem from 13 days old plantlets were taken and inoculated on the Murashige and Skoog (MS) medium. All of this procedure was performed in sterilized conditions. Inoculated flasks were then kept in a climatic growth chamber (light/dark period of 16/8 h) at 25 ± 1 °C temperature for 30 days.

### 2.6. Oxidative Stress Analysis

#### 2.6.1. ROS Production

ROS formation as well as membrane lipid peroxidation were conducted as described in Hano et al. [32].

#### 2.6.2. Protein Carbonyl Content

Total proteins were extracted as described in Hano et al. [32] and their carbonylation content was determined by ELISA method (OxiSelect™ Protein Carbonyl ELISA Kit, Cell Biolabs, San Diego, CA, USA). Briefly, the cells were grinded in liquid nitrogen using a mortar and pestle, and then homogenized with a 10 mM Tris-HCl (pH 7.4) containing 3 mM MgCl_2_ and 2 mM β-mercaptoethanol. The homogenates were centrifuged at 10,000× *g* for 10 min and the total protein content was determined using the Quant-iT Protein Assay Kit (Invitrogen, Thermo Fisher, Illkirch, France) using the Qubit fluorometer. The protein carbonylation level was determined as described by the manufacturer’s instructions, by measuring the absorbance value at 405 nm, and the relative protein carbonylation level was expressed as a percentage relative to the A405 value measured for the control.

#### 2.6.3. DNA 8-oxo-Guanine Content

DNA was extracted by the cetyl-trimethyl-ammonium bromide (CTAB) method [33]. DNA contents were determined using the Quant-iT DNA BR Assay Kit (Invitrogen, Thermo Fisher, Illkirch, France) using the Qubit fluorometer (Invitrogen, Thermo Fisher, Illkirch, France). The 8-oxoGuanine level determined by the ELISA method (Oxiselect oxidative DNA damage ELISA kit, Cell Biolabs, San Diego, CA, USA) as described by the manufacturer’s instructions by measuring the absorbance value at 405 nm, and the relative protein carbonylation level was expressed as a percentage relative to the A405 value measured for the control.

### 2.7. Enzymatic Antioxidant Response

#### 2.7.1. Enzyme Extraction

Protein extraction was performed as described above for the protein carbonylation assay. The protein content determined using the Quant-iT Protein Assay Kit (Invitrogen, Thermo Fisher, Illkirch, France).

#### 2.7.2. Peroxidase Activity

Peroxidase (POD) activity was determined performed as described by Lagrimini [34]: 20 μL of guaiacol (1 M), 40 μL of 50 mM potassium phosphate buffer (pH 7), 20 μL of fresh sample extract, 20 μL of H_2_O_2_ (27.5 M) and 100 μL of dH_2_O were mixed to prepare the reaction mixture. The absorbance value was recorded after 20 s with a microplate reader at 470 nm to determine the POD activity.

#### 2.7.3. Superoxide Dismutase Activity

Superoxide dismutase (SOD) activity was determined as described by Giannoplolitis and Ries [35]: 20 μL methionine (130 mM), 20 μL nitroblue tetrazolium (NBT) (0.75 mM), 20 μL ethylenediaminetetraacetic acid (EDTA) (1 mM), 78 μL phosphate buffer (50 mM, pH 7), 2 μL riboflavin (0.02 mM) and 60 μL fresh sample extract were mixed and then kept under florescent light for 7 min. Then, the absorbance value was determined at 660 nm using a microplate reader to determine the SOD activity.

### 2.8. Non-Enzymatic Antioxidant Response

#### 2.8.1. Methanolic Extracts Preparation

Extracts were prepared as described previously [28]: the fine powder was made by grinding the dried biomass of plantlets and callus and then 500 mg of the powdered sample was taken in 500 mL methanol to vortex and sonicate (Elmasonic E 30 H, Elma Schmidbauer GmbH, Singen, Germany) for 30 min. The prepared extract was centrifuged (Spectrafuge™ 24D microcentrifuge, Labnet International, Corning, NY, USA) for 15 min at 10,000 rpm to collect supernatant.

#### 2.8.2. Free Radical Scavenging Activity 

Free radical scavenging activity (FRSA) was determined as described by Lee et al. [36]: 20 μL extract sample and 180 μL of the 2,2-Diphenyl-1-picrylhydrazyl (DPPH) solution were mixed in a 96-well microplate. The plate was then placed in the darkness for 1 h and the absorbance was determined at 517 nm using the microplate reader. The formula used for calculating the FRSA was: FRSA (%) = 100 × (1 − AE/AD), with AE is the mixture absorbance at 517 nm, while AD represents only the DPPH absorbance.

#### 2.8.3. Total Phenolic Content (TPC)

TPC was determined as described by Singleton and Rossi [37]: 20 µL extract sample, 90 µL Na_2_CO_3_ and 90 µL Folin–Ciocalteu reagent (Sigma-Aldrich, Saint-Quentin Fallavier, France) were mixed to prepare the reaction mixture, an absorbance at 630 nm was measured and the TPC was determined in mg/g dry weight (DW) of gallic acid equivalent, using a 5-point calibration curve (0–40 µg/mL; gallic acid; *R*^2^ = 0.998).

#### 2.8.4. Total Flavonoid Content (TFC)

TFC was determined as described by Ul-Haq et al. [38]: 20 µL extract, 10 µL aluminum chloride, 10 µL potassium acetate and 160 µL distilled water were mixed and then incubated for 30 min, then absorbance at 415 nm was measured and the TFC was determined in mg/g DW quercetin equivalent using a 5-point calibration curve (0–40 µg/mL; quercetin; *R*^2^ = 0.998).

#### 2.8.5. HPLC Analysis

Contents of lignans and neolignans were determined as described previously [39,40,41]: 500 mg of lyophilized cells were extracted with a 20 mL of 80% (*v/v*) aqueous methanol solution by sonication (USC1200TH (Prolabo, Sion, Switzerland) during 1h, at 45 kHz and 25 °C. The extract was then centrifuged at 3000 rpm for 15 min, the resultant supernatant was evaporated into dryness at 40 °C, prior to aglycone released by β-glucosidase (5 unit/mL, Sigma, Saint Quentin Fallavier, France) in citrate-phosphate buffer (1 mL, pH 4.8). Before injection, the extract was filtered with a syringe filter (0.45 µm, Millipore, Molsheim, France). The reverse phase high-performance liquid chromatography (RP-HPLC) system composed of Varian Prostar 230 pump, Degasser (Metachem Degasit), Varian Prostar 335 PAD, Varian Prostar 410 auto sampler and driven with the Galaxie software (v1.9.3.2) and RP-18 column (Purospher (Merck, Saint Quentin Fallavier, France); 5 μm; 250 × 4 mm) were used for the separation procedures at 35 °C according to a previous method [40]. Calibration curves were used for quantifying the lignans and neolignans and the results were represented in mg of glucoside equivalent (lariciresinol diglucoside, secoisolariciresinol diglucoside, guaiacylglycerol-*β*-coniferyl alcohol and dehydrodiconiferyl alcohol glucoside), which was equal to the mass per gram of the DW. The internal standard used (*o*-coumaric acid), calibration curves, limits of detection and quantification as well as the validation of the method were described in Anjum et al. [41].

### 2.9. Statistical Analysis

The biological replicates used in the experiment were nine for the seedlings, and three for the callus cultures. Moreover, all experiments had three technical replicates. Data were statistically analyzed using Origin (Windows v8.1, Northampton, MA, USA) and Microsoft Excel software (Albuquerque, NM, USA).

## 3. Results and Discussion

### 3.1. Comparative Impact of ZnONP Applications on Flax Seedlings vs. Callus Growth

**Figure 1:**
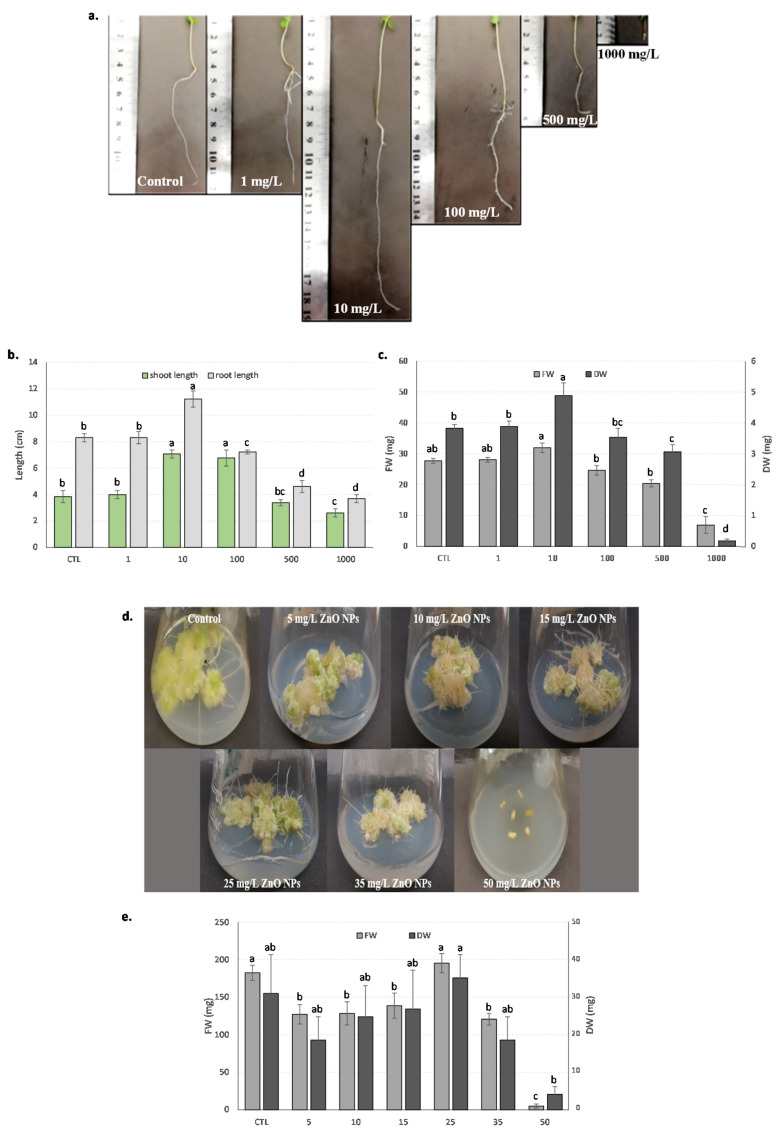
Effect of zinc oxide nanoparticles (ZnONPs) on the seedlings and the stem-derived callus of *L. usitatissimum*. (**a**) The morphological evaluation with the centimeter scale to comprehend the root and shoot length of the *L. usitatissimum* seedlings at different concentrations of ZnONPs; (**b**) the trends in the root and shoot length of the *L. usitatissimum* seedlings at different concentrations of ZnONPs (the x axis represents the control (CTL) and the ZnONP treatment (at 1, 10, 100, 500 and 1000 mg/L ZnONPs, respectively) conditions); (**c**) the biomass (fresh and dry weight) accumulation of *L. usitatissimum* seedlings at different concentrations of ZnONPs (the x axis represents the control (CTL) and the ZnONP treatment (at 1, 10, 100, 500 and 1000 mg/L ZnONPs, respectively) conditions); (**d**) the morphological evaluation of the effect of the different concentrations of ZnONPs on the stem-induced callus of *L. usitatissimum*; (**e**) the trends in the biomass (fresh and dry weight) accumulation of the stem-induced callus of *L. usitatissimum* at different concentrations of ZnONPs (the x axis represents the control (CTL) and the ZnONP treatment (at 5, 10, 15, 25, 35, 50 mg/L ZnONPs, respectively) conditions). Different letters indicate significant differences (*p* < 0.05).

Flax seedlings and callus deriving from stem explants were also inoculated with ZnONPs provided in different concentrations (0–1000 mg/L for the seedlings vs. 0–50 mg/L for the callus) to investigate the effect of ZnONPs on both culture systems. Morphological aspects as well as growth evaluation results are presented in Figure 1.

All the inoculated seeds presented radicle development of varying length, i.e., ranging from 2.62 ± 0.31 cm (for 1000 mg/L ZnONPs) to 7.08 ± 0.34 cm (for 10 mg/L ZnONPs) with 3.85 ± 0.46 cm for the control, showing a seed germination efficiency of varying levels under the different concentrations of ZnONPs. With a concentration of ZnONPs above 10 mg/L, the higher the concentration of NPs, the lower the radicle length. NPs have a large surface area which has resulted in the increased sensitivity of the radicle to toxicants [42]. This inhibitory effect on seed germination may be because of the very small size of NPs and the dissolution power of ZnO to Zn^2+^ ions [43]. The investigation of the L. usitatissimum plantlets revealed a stimulatory effect of ZnONPs at a concentration of 10 mg/L with a 37% increase in the overall length and a notable inhibitory effect was found at a 1000 mg/L concentration with 87% retarded growth along length (Figure 1a). Similar trends for the shoot length variation were recorded (i.e., with shoot length values ranging from 3.69 ± 0.38 cm (for 1000 mg/L ZnONPs) to 11.23 ± 0.62 cm (for 10 mg/L ZnONPs); with 8.31 ± 0.34 cm for control). In addition to the measured values of the shoot and root length, both the fresh weight (FW) and DW showed similar increasing and decreasing trends as shown in Figure 1b,c, respectively. This positive stimulatory effect was recorded for seedling growth at a 10 mg/L non-lethal concentration of ZnONPs.

Similar stimulating effects of nanomaterials on seedlings from different species have been described, as for example in changbai larch [44] and soybean [45]. The potential positive impact of ZnONPs for increasing crop production has been also reported [46]. Along with the increasing concentration of ZnONPs, the length of the root and the shoot decreased significantly, with roots being more affected by the direct contact with NPs. The negative effects of the NPs on root elongation may have been due to the non-porous nature of the medium agar, water logging and the less available dissolved oxygen [43]. Root development was drastically reduced at a concentration of 1000 mg/L of ZnONPs. This observation shows that mitosis may be adversely affected by the presence of NPs, as previously documented [47]. This substantial decrease in the plantlet growth is consistent with previous wheat field studies, where the application of excessive NPs resulted in reduced plantlet stature and distorted plantlet physiology [48].

Similarly, the callus response was dependent on ZnONP dosage, and an overall inhibiting effect was found as the concentration of NPs increased. Interestingly, impressive rooting was observed at mild concentrations (35 mg/L) of ZnONPs, as shown in Figure 1d. The presence of induced rooting at higher concentrations of NPs could be explained by the way zinc acts at a specific concentration to induce the production of auxins in plants [49]. Moreover, zinc may also act like an activator or a cofactor for the production of particular enzymes that play an important role in rooting [50]. Maximum biomass (FW: 195.32 g/L, DW: 34.46 g/L) was reported using 25 mg/L ZnONPs, which was 3.8% and 11.11% higher than the control, respectively (Figure 1e). This increase in biomass was mainly observed because of excessive rooting followed by spontaneous decline along with further increase in NPs concentration.

### 3.2. Oxidative Stress-Induced ZnONP Applications on Flax Seedlings vs. Callus

**Figure 2:**
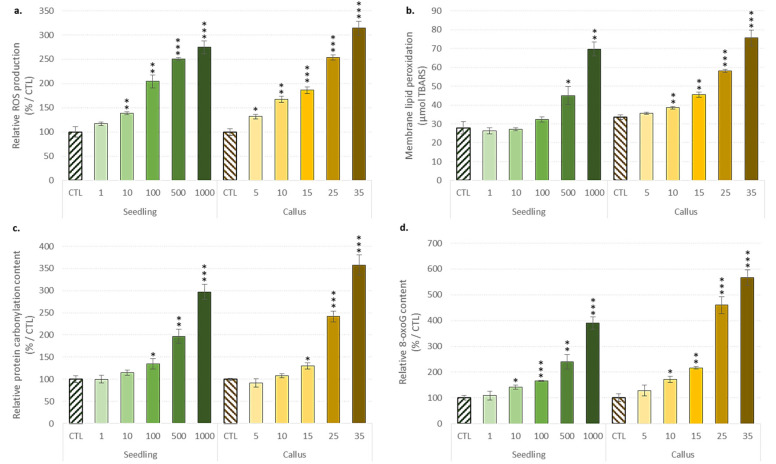
Evaluation of the oxidative stress induced by the ZnONP application on the seedlings and the stem-derived callus of *L. usitatissimum*. (**a**) The reactive oxygen species (ROS) formation (relative fluorescence unit); (**b**) the membrane lipid peroxidation (µmol thiobarbituric acid-reactive substances; TBARS); (**c**) the protein carbonylation formation (A_405_/10 µg protein); (**d**) the 8-oxo guanine (8-oxo-G) formation (A_405_/10 µg protein). Values are means ± SD of three independent experiments; * *p* < 0.05, ** *p* < 0.01, *** *p* < 0.001.

Several methods have been developed to detect and quantify ROS in plant tissues or extracts, including the fluorescent dihydrofluorescein diacetate probe [51]. Using this fluorescent probe, the relative quantification of hydrogen peroxide concentrations revealed an induction of oxidative stress, observed as a consequence of the ZnONP applications on the two different flax biological systems (Figure 2).

A gradual increase in ROS production was observed in both flax cultivation systems, with a significant increase in oxidative stress measured for ZnONP concentrations reaching values of 10 mg/L and 5 mg/L for the flax seedlings and the stem-derived callus, respectively (Figure 2a). As a consequence of this oxidative stress, increased membrane lipid peroxidation, protein carbonylation and 8-oxo-guanine DNA oxidative damages were detected (Figure 2b–d).

Hydrogen peroxide is a major ROS, often known to be a signaling molecule involved in many stress adaptations in plants [51,52]. However, uncontrolled ROS production, which occurs when ROS levels exceed the plant’s antioxidant defense capability, may result in damage to the integrity and functions of the membrane, the disruption of the metabolic pathway through the inactivation of the enzyme, or an increase in the genome instability due to DNA mutations affecting different physiological processes and finally, cell viability [53,54]. Increase in the membrane lipid peroxidation induced by NP applications has already been reported [55,56]. The membrane lipid peroxidation degree has been proposed to be linearly associated with metabolic perturbations [57]. Higher membrane integrity also allows more tolerant plant species or systems to maintain homeostasis through efflux mechanisms that prevent and/or reduce the entry of NPs into cells, as observed with electrolyte leakage perturbations reported [58]. Protein-level oxidative damage has been suggested to hinder the detoxification process and reduce the plant tolerance to stress [57,59]. The exposure to NPs induced DNA damage [60], indicating possible genotoxic effects that could lead to cell death [53,54]. Here, we have observed that this oxidative stress could also lead to the formation of 8-oxo-guanine, one of the major mutagenic DNA damages that could significantly impair gene expression and function. Besides all these negative impacts, ROS is also a key secondary messenger involved in various developmental processes such as seed germination [61], root development and response to (a)biotic stress [62]. Here, it can be assumed that a moderate increase in ROS production induced by the use of sublethal ZnONPs could result in the observed stimulation of root growth and/or de novo formation in flax seedlings and in vitro stem-derived callus.

### 3.3. Enzymatic Antioxidant Response of Flax Seedlings vs. Callus Challenging with ZnONPs

**Figure 3:**
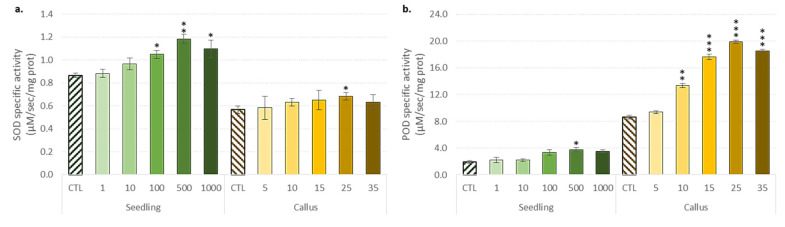
Effect of the different concentrations of ZnONPs on the antioxidant enzymes. (**a**) The superoxide dismutase activity (SOD) and (**b**) the peroxidase activity (POD) in the seedlings and the stem-derived callus of *L. usitatissimum*. Values are means ± SD of three independent experiments; * *p* < 0.05, ** *p* < 0.01, *** *p* < 0.001.

Both superoxide dismutase (SOD) and peroxidase (POD) antioxidant enzyme activities were then evaluated (Figure 3).

Responses to the antioxidant enzyme activity here depend not only on the cultivation system considered and the concentrations of ZnONPs, but also more intriguingly on the type of enzyme. The activation of both SOD and POD activities was observed in seedlings reaching a maximum value of 500 mg/L of ZnONPs. The same increasing trend was observed for the stem-derived callus with a maximum activation of 25 mg/L ZnONPs. However, the activation of SOD activity was significantly higher than that observed for the POD activity in flax seedlings, while the opposite trend was observed for the stem-derived callus (Figure 3a,b). We also noted a sudden decline in values at higher NP concentrations (Figure 3). This sudden drop in enzymatic reactions (for concentrations above 500 mg/L for seedlings and 25 mg/L for callus ZnONPs) may result from a loss in the ability to withstand the impact of ROS, as confirmed by the observed increase in oxidative damage (Figure 2).

The antioxidant response to ROS scavenging is a means of avoiding potential oxidative damage caused by the application of NPs. To do so, plants developed a set of antioxidant defense systems that rely on enzymatic and/or non-enzymatic mechanisms [53,54]. Since ROS was often used by plants as a second messenger for the signal transduction of multiple physiological reactions, ROS cannot be completely scavenged. As a result, plants developed a very complex array of mechanisms to maintain ROS at a permissive level, and to prevent the harmful effects observed at excessive concentrations. Plants may activate of a variety of antioxidant enzymes to detoxify excessive ROS output, to cope with oxidative stress [63]. The activation of antioxidant enzymes in response to NP applications has been reported in the seedlings of *Ricinus communis* [64] and *Arabidopsis thaliana* plantlets [65], as well as in vitro shoot cultures of *Vanilla planifolia* [66]. SOD is a well-known metalloprotein that catalyzes the dismutation of the superoxide anion to H_2_O_2_ [67], and its activation may protect plants from oxidative damages [68,69]. Similarly, POD is the main enzyme involved in the elimination of H_2_O_2_ [67]. Here, the observed differential induction of the antioxidant enzyme has already been described in various physiological processes [70]. Increasing the enzymatic antioxidant response may counteract the oxidative stress induced by the use of NPs, as previously reported [46]. In the future, these enhanced enzymatic and non-enzymatic responses could also serve as a tool for improving the productivity of flax, as already reported in *Capsicum annum* [71].

### 3.4. Non-Enzymatic Antioxidant Response of Flax Seedlings vs. Callus Challenging with ZnONPs

**Figure 4:**
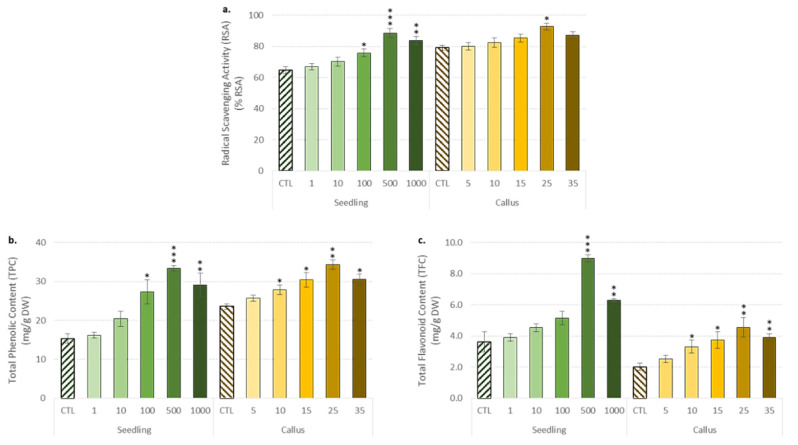
Influence of the different concentrations of ZnONPs on non-enzymatic antioxidant response in the seedlings and the stem-derived callus of *L. usitatissimum.* (**a**) The free radical scavenging activity (FRSA); (**b**) the total phenolic content (TPC); and (**c**) the total flavonoid content (TFC). Values are means ± SD of three independent experiments; * *p* < 0.05, ** *p* < 0.01, *** *p* < 0.001.

In addition to the enzymatic antioxidant system, non-enzymatic antioxidants are of particular interest to control oxidative stress due to their high antioxidant properties. Flax is a rich source of antioxidant phenylpropanoid-derived compounds [3,6]. The next step was to record trends in free radical scavenging activities (FRSA, Figure 4a), as well as TPC (Figure 4b) and TFC (Figure 4c) in both flax seedlings and in vitro callus treated with ZnONPs.

**Figure 5:**
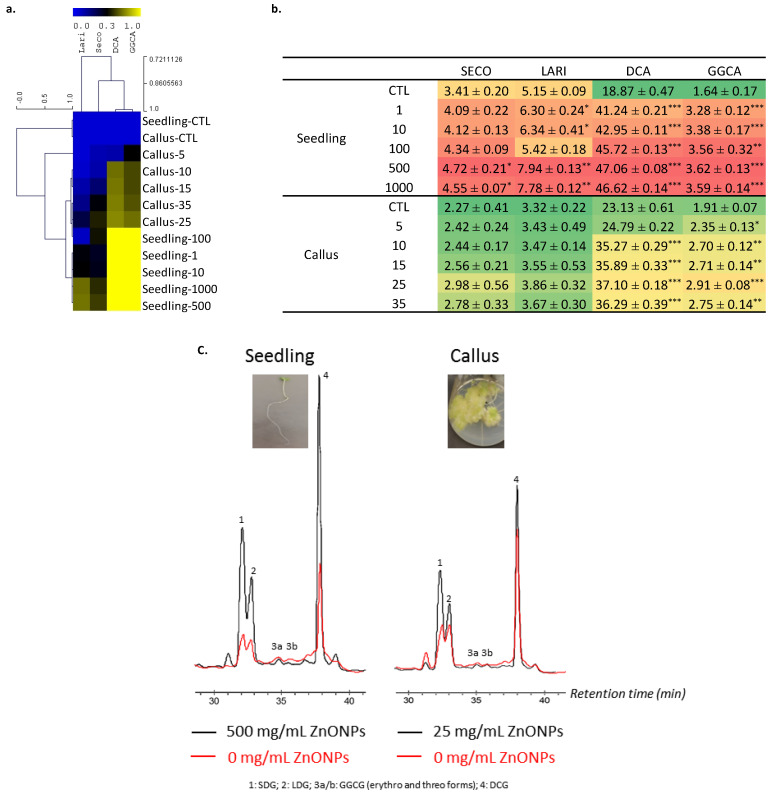
Influence of the different concentrations of ZnONPs on (neo)lignans biosynthesis in the seedlings and the stem-derived callus of *L. usitatissimum.* (**a**) The hierarchical clustering analysis of the (neo)lignans production; (**b**) the actual values of the (neo)lignans accumulation (in mg/g DW); (**c**) the typical HPLC chromatograms of the control and the ZnONP-treated flax seedling and stem-derived callus. Values are means ± SD of three independent experiments; * *p* < 0.05, ** *p* < 0.01, *** *p* < 0.001.

A similar trend was recorded for TPC and TFC with a gradual increase as a function of ZnONP concentration applied reaching a maximal value (at 500 mg/L for seedlings vs. 25 mg/L for callus) before decreasing above this critical value (Figure 4a,b). The increase in TPC and TFC in flax the plantlets was to combat the oxidative stress induced by NPs, as previously reported [15]. In good agreement with this observation, the TPC and TFC increases were well correlated with the non-enzymatic radical scavenging activity (Figure 4a). ZnONPs act as abiotic elicitors, affecting plant physiology and secondary metabolite production. This is because they trigger the upregulation of potential antioxidant metabolism [72,73]. Analogous supporting results have been successfully reported in *Brassica nigra* [74]. Targeted HPLC analysis focusing on the main compounds accumulated in flax confirmed the effect of ZnONP application on the activation of the (neo)lignan pathway (Figure 5a–c).

A global increase was observed, for the most abundant common (neo)lignans accumulated in both the seedlings and the callus cultures: secoisolariciresinol diglucoside (SDG, 1) and lariciresinol diglucoside (LDG, 2) for lignans, *erythro*- and *threo*-guaiacylglycerol-β-coniferyl alcohol ether glucoside (GGCG, 3a-b) and dehydrodiconiferyl alcohol glucoside (DCG, 4) (Figure 5c).

Here, the stimulatory effect of ZnONPs at different concentrations on lignans (secoisolariciresinol diglucoside; SDG, lariciresinol diglucoside; LDG) and neolignans (dehydrodiconiferyl alcohol glucoside; DCG and Guaiacylglycerol-β-coniferyl alcohol ether glucoside; GGCG) biosynthesis was also explored. The ZnONPs resulted in a higher accumulation of SDG (4.72 mg/g DW), LDG (7.94 mg/g DW), DCG (47.06 mg/g DW) and GGCG (3.62 mg/g DW) in plants at a 500 mg/L concentration, which was 27.7, 35.1, 59.9 and 54.6% higher than in the control, respectively (Figure 5a,b). However, higher concentrations of ZnONPs decreased the production of lignans and neolignans. These outcomes can be explained as a protective action against reactive oxygen species (ROS) induced by the accessibility of NPs. Moreover, the stimulatory effect on dietary lignans; SDG and LDG, and neolignans; DCG and GGCG, is because they act as antioxidants and free radical scavengers, which could be employed in medicinal science, mainly for the purpose of reducing cancer risk [75]. Our study suggested that the ZnONPs have a significant role in augmenting the secondary metabolite production pathways, as previously testified in *Bacopa monnieri* [71].

ZnONPs also increased the biosynthesis of SDG (2.98 mg/g DW), LDG (3.86 mg/g DW), DCG (37.10 mg/g DW) and GGCG (2.91 mg/g DW) in the callus cultures at 25 mg/L concentration, which was 23.8, 14.0, 37.7 and 34.3% higher than control, respectively (Figure 5a,b). Callus treated with ZnONPs increased the production of metabolites as a necessary part of the antioxidant mechanism. Comparable results were recorded in *Stevia rebaudiana*-treated callus cultures [76]. The increased level above 35 mg/L of ZnONPs retarded the callus ability to withstand ROS and the callus biomass reduced significantly, which is in harmony with the studies of Mittler et al. [77] and Stadtman and Oliver [78]. The reason behind the excess metabolite production is still not clear but various reports have confirmed ZnONPs as potential abiotic elicitors in in vitro cultures for medicinally important metabolite production [79,80]. Callus cultures at higher concentration of ZnONPs had a reduced biomass when compared to the control because, above certain level, Zn becomes toxic and denatures proteins at the extent of retarding the growth and development of plant cells [81]. Zn toxicity hinders growth, biochemical processes and catalytic efficiency and may possibly completely halt growth, as observed in this study at a 50 mg/L concentration of ZnONPs [82]. This is why it is very essential to dispose of industrial wastes and sludge containing metal oxide NPs with proper care, because these waste waters can enter agriculture fields [83].

## 4. Conclusions

To summarize the current research, it can be stated that the presence of ZnONPs in a medium at different concentrations significantly enhances antioxidant responses and non-enzymatic antioxidants in both *L. usitatissimum* seedlings and stem-derived callus. In addition to the enhanced antioxidant effect of ZnONPs on stem-derived callus, excessive rooting can be observed at lower concentrations of NPs, which could be used as a means of producing valuable metabolites for various medicinal purposes. In both systems, dose-dependent ROS production was recorded, resulting in oxidative damage (lipids, proteins and DNA) with a more pronounced toxic effect in in vitro cultures. A differential induction of SOD (in seedlings) vs. POD (in vitro cultures) enzymes was noted, whereas the activation of secondary metabolite accumulation and radical-scavenging activity appeared as common features. Altogether, the results from this study constitute a first comprehensive view of the impact of ZnONPs on the oxidative stress and antioxidant responses in seedlings vs. in vitro cultures. Moreover, from a biotechnological point of view, the increased production of lignans and neolignans also suggests that their metabolic engineering is possible for medical and health purposes. This study also shows that ZnONP elicitation can be used effectively for the large-scale biogenesis of important metabolites in both seedlings and in vitro cultures of *L. usitatissimum* to address various health and commercial concerns.
